# Remibrutinib (LOU064) inhibits neuroinflammation driven by B cells and myeloid cells in preclinical models of multiple sclerosis

**DOI:** 10.1186/s12974-023-02877-9

**Published:** 2023-08-26

**Authors:** Barbara Nuesslein-Hildesheim, Enrico Ferrero, Cindy Schmid, Catherine Huck, Paul Smith, Sarah Tisserand, Joelle Rubert, Frederic Bornancin, Denis Eichlisberger, Bruno Cenni

**Affiliations:** 1 Novartis Institutes for Biomedical Research, Basel, Switzerland; 2 Recludix Pharma, San Diego, USA

**Keywords:** Multiple sclerosis, BTK, Remibrutinib, LOU064, Autoimmunity, Neuroinflammation

## Abstract

**Background:**

Bruton’s tyrosine kinase (BTK) is a key signaling node in B cell receptor (BCR) and Fc receptor (FcR) signaling. BTK inhibitors (BTKi) are an emerging oral treatment option for patients suffering from multiple sclerosis (MS). Remibrutinib (LOU064) is a potent, highly selective covalent BTKi with a promising preclinical and clinical profile for MS and other autoimmune or autoallergic indications.

**Methods:**

The efficacy and mechanism of action of remibrutinib was assessed in two different experimental autoimmune encephalomyelitis (EAE) mouse models for MS. The impact of remibrutinib on B cell-driven EAE pathology was determined after immunization with human myelin oligodendrocyte glycoprotein (HuMOG). The efficacy on myeloid cell and microglia driven neuroinflammation was determined in the RatMOG EAE. In addition, we assessed the relationship of efficacy to BTK occupancy in tissue, ex vivo T cell response, as well as single cell RNA-sequencing (scRNA-seq) in brain and spinal cord tissue.

**Results:**

Remibrutinib inhibited B cell-dependent HuMOG EAE in dose-dependent manner and strongly reduced neurological symptoms. At the efficacious oral dose of 30 mg/kg, remibrutinib showed strong BTK occupancy in the peripheral immune organs and in the brain of EAE mice. Ex vivo MOG-specific T cell recall response was reduced, but not polyclonal T cell response, indicating absence of non-specific T cell inhibition. Remibrutinib also inhibited RatMOG EAE, suggesting that myeloid cell and microglia inhibition contribute to its efficacy in EAE. Remibrutinib did not reduce B cells, total Ig levels nor MOG-specific antibody response. In brain and spinal cord tissue a clear anti-inflammatory effect in microglia was detected by scRNA-seq. Finally, remibrutinib showed potent inhibition of in vitro immune complex-driven inflammatory response in human microglia.

**Conclusion:**

Remibrutinib inhibited EAE models by a two-pronged mechanism based on inhibition of pathogenic B cell autoreactivity, as well as direct anti-inflammatory effects in microglia. Remibrutinib showed efficacy in both models in absence of direct B cell depletion, broad T cell inhibition or reduction of total Ig levels. These findings support the view that remibrutinib may represent a novel treatment option for patients with MS.

**Supplementary Information:**

The online version contains supplementary material available at 10.1186/s12974-023-02877-9.

## Background

Multiple sclerosis (MS) is a chronic, autoimmune demyelinating disease of the central nervous system (CNS) that is associated with severe morbidity and impaired quality of life [1]. The pathological hallmark of MS is the lesion, being in the majority of cases a sharply demarcated demyelinated area in the CNS white matter that expresses variable degrees of inflammation, axonal injury, gliosis, and myelination [2]. Lesion formation involves the synergistic action of cellular and humoral autoimmune reactions directed against components of the myelin sheath [3]. Ultimately this autoimmune response causes damage to the myelin sheath, oligodendrocyte death and axonal loss.

Both B and T cells are critically important in the immune-mediated pathogenesis of MS. Specifically, B cells are contributing to the immune-mediated histopathology, are present in areas of demyelination, in the cerebrospinal fluid (CSF) of MS patients as well as in chronic plaques [4, 5]. Myelin-reactive memory B cells have been found in the peripheral blood of MS patients and it is thought that these autoreactive B cells contribute to MS pathogenesis as antigen presenting cells (APC) activating CD4 + T cells, as well as by their secretion of proinflammatory cytokines and autoreactive antibodies [5–7].

The clinical efficacy of anti-CD20 B cell-depleting therapies such as rituximab, ocrelizumab, and ofatumumab validate the crucial role of B cells in MS [8–11].

Bruton’s tyrosine kinase (BTK) is a cytoplasmic tyrosine kinase selectively expressed in a subset of immune cells—B cells, monocytes/macrophages, platelets, mast cells and basophils, but not in T cells or mature plasma cells [12]. Inborn BTK deficiency is a cause for the primary immunodeficiency X-linked agammaglobulinemia (XLA), a condition that if untreated is associated with an increased bacterial infection risk [13]. Today XLA is treated by immunoglobulin replacement therapy, suggesting that the main risk factor for infections is the absence of protective immunoglobulins rather than the deficiency in cellular BTK function [14]. The role of BTK appears not to be critical for B cell survival once the B cells have developed, suggesting that BTK inhibition after infancy may not lead to acute B cell and antibody depletion [15]. This could be a clinically relevant differentiating factor for the therapy with BTKi compared to long-term B cell-depleting agents.

BTK is indispensable for signaling through B cell antigen receptor (BCR), as well as the Fc epsilon receptor (FCER1) and the activating Fc gamma receptors (FCGR) [16]. Inhibition of BTK may therefore be an attractive therapeutic concept to treat various autoimmune and chronic inflammatory diseases, including MS [17, 18]. Indeed, genetic BTK deficiency reduced mouse EAE [19], and more recently pharmacologic BTK inhibition showed efficacy in a preclinical EAE model [20], as well as in clinical MS study [21]. This is in line with the central role of B cells in MS established by the clinical efficacy of B cell-depleting agents [22].

Microglia are the CNS-resident phagocytes and express BTK [23, 24]. Like other cell types of the myeloid lineage, microglia may also contribute to a local inflammatory response by responding to pathogenic triggers like autoantibodies or innate immune stimuli. This local inflammatory response may exacerbate pathology in MS and may offer a mechanistic intervention that goes beyond direct B cell depletion by injectable anti-CD20. The emerging role of BTK in myeloid cells of the CNS like microglia further supports a potential benefit of BTK inhibition in MS, i.e. by dampening microglial activation, which drives injury in MS [23, 24].

Remibrutinib is a selective, potent, covalent inhibitor of BTK [25]. Owing to its specific binding mode to BTK, remibrutinib showed very high selectivity and due to its covalent mode of action it showed highly potent BTK inhibition at very low exposures in preclinical models [26]. First clinical trials indicate a very attractive profile that is characterized by effective inhibition of BTK-dependent pathways and a very promising safety profile at the current stage of clinical development [27, 28]. Remibrutinib has shown in vitro inhibition of BCR-dependent B cell APC function and FcGR-dependent proinflammatory response in myeloid cells [26].

In the present studies, we assessed the effects of remibrutinib in preclinical models of MS with a focus on two clinically relevant pathomechanisms—inhibition of the neuropathogenic APC function of B cells and of proinflammatory myeloid cells. Mouse EAE models show qualitative differences in the cell types driving the pathology [29]. In C57BL/6 mice variants of myelin oligodendrocyte glycoprotein (MOG)-induced EAE have been described, depending on whether the human (HuMOG) or rat (RatMOG) recombinant protein sequences are used for immunization [30, 31]. Minor amino acid residue differences result in HuMOG-induced EAE being B cell dependent as the main APC, while RatMOG-induced EAE is B cell independent and requires dendritic cells as the dominant APC [32]. Given the role of BTK in B cells and the sensitivity of the HuMOG model to anti-CD20 depletion [33], we selected this model to asses B cell-dependent remibrutinib efficacy. As the RatMOG model has been shown to be less B cell dependent, we chose it in a second stage to better discern remibrutinib effects mediated by other cell types like myeloid cells and microglia.

Remibrutinib showed efficacy in EAE model indicating a dual mechanism based on inhibition of B cells, as well as of myeloid cells and microglia.

## Methods

### Animal experiments

Mouse experiments were performed in accordance to Novartis and Swiss animal welfare regulations. Mice were specific pathogen free and maintained in individually ventilated cages with a 12/12 h light/dark cycle. For induction of EAE female C57BL/6 mice of matched age and weight (Harlan, Switzerland or Envigo, Switzerland) were immunized as previously described [32–34]. Briefly, mice received a subcutaneous injection of recombinant human MOG (amino acids 30–149, produced in house) or recombinant rat MOG (amino acids 28–152, produced in house) emulsified in complete Freund adjuvant (Sigma, Switzerland) for huMOG EAE and for rat MOG EAE, respectively. Pertussis toxin (Fluka, Switzerland) was administered intraperitoneally on day 0 and 2. Disease was monitored daily using a scoring system (0: normal appearance; 1: complete tail paralysis; 2: unilateral partial hind limb paralysis; 3: complete bilateral hind limb paralysis; 4: quadriplegia; 5: death) [35]. Remibrutinib was dosed as suspension in 0.5% methylcellulose, 0.5% Tween 80 in water. Dosing of remibrutinib or vehicle by gavage started from the time of immunization. Scoring was performed by an operator unaware of the treatment groups. Mice were killed at study end by inducing deep anesthesia using isoflurane. Tissues were perfused with saline to avoid blood contamination of sampled organs and blood/CSF samples were taken prior to perfusion. For CSF withdrawal, skin and outer layer of muscles at the neck are cut with a scalpel blade, further muscles are separated from the skull and pulled back using cotton swabs. The dura mater is perforated with the tip of an injection needle and the emerging CSF is collected with a pipette. CSF withdrawal was done under terminal anesthesia.

### Serum NfL assay

Serum NfL levels were determined with a modified MSD (Meso Scale Discovery) assay using a cross-reactive anti-human NfL capture antibody (Uman Diagnostics, Sweden) and detection with a biotinylated anti-human IgG antibody (Uman Diagnostics, Sweden).

### T cell response analysis

Ex vivo antigen recall proliferation responses were studied using splenocytes and draining inguinal lymph node cells isolated on day 8 after immunization with HuMOG and before onset of disease and under remibrutinib treatment. Isolated cells were counted and equal numbers of cells were added to round-bottom well plates coated either with HuMOG peptide or antiCD3/antiCD28 (R&D Systems, USA and BD, USA, respectively). After 72 h [3H]-thymidine (PerkinElmer, USA) was added and 16 h later cells were harvested and incorporated radioactivity was measured in a MicroBetaTrilux counter (PerkinElmer, Switzerland).

### Cell subset and intracellular cytokine analysis by flow cytometry

In the same 8-day HuMOG immunization study cells were analyzed by flow cytometry for changes in subpopulations and cytokine response. Isolated cells were counted and equal number used for subpopulation analysis gated on live lymphocytes by forward/side scatter. Cells were stained with an antibody panel including CD3 (BD BioSciences, Switzerland), CD4 (BD BioSciences, Switzerland) and CD19 (BioLegend, USA).

For cytokine secretion analysis cells were stimulated with PMA/ionomycin for 4 h in presence of brefeldin A. Then cells were fixed, permeabilized and stained with antibodies for CD3, CD4, interferon γ and IL-17 (all BD BioSciences, Switzerland) and analyzed by flow cytometry by gating on live lymphocytes by forward/side scatter (Additional file 1: Fig. S3).

### BTK occupancy

BTK occupancy in tissue was analyzed as described [26] with immunoassays for free BTK protein (i.e. not covalently occupied by compound) and total BTK protein using the MSD platform. For free BTK measurements, a streptavidin-coated MSD assay plate was incubated with a biotinylated covalent BTK probe [36], then samples were added to allow binding of the unoccupied free BTK to the plate-bound probe. Plate-bound BTK was detected with a labeled anti-BTK antibody (D3H5, Cell Signaling Technology, USA). For total BTK measurements, an MSD assay plate was coated with D3H5 anti-BTK to capture total BTK. A labeled anti-BTK antibody (#53, BD Biosciences, Switzerland) was then used to detect captured BTK. The signals from both assays were calibrated against standard curves of recombinant BTK protein. The respective free BTK levels for each sample were normalized to the total BTK level in the same sample and these ratios were expressed as percentage of the vehicle control samples.

### Single cell transcriptomic analysis

Mice from a dedicated ratMOG EAE study were killed at d19 or d29 under deep isoflurane anesthesia, then perfused with cold saline to avoid blood contamination of spinal cord and brain.

Entire brains and spinal cords were removed and single cells prepared by dissociation with adult brain dissociation kit in a GentleMACS apparatus (Miltenyi Biotec, Germany) as described [37]. The two timepoints were preselected as they were likely to reflect peak of clinical scores and the terminal phase.

The 10X Genomics Chromium raw sequencing reads were processed with Cell Ranger. All subsequent analyses were carried out in R v4.1 and Bioconductor v3.14, as described [38]. One sample was excluded from the analysis due to the low number of unique molecular identifiers (UMIs) per cell and suspected contamination with ambient RNA. Adaptive-threshold quality control was used to remove low-quality cells with low library size, low number of detected genes, high proportion of mitochondrial reads or doublets. In total, 76,287 cells passed quality control.

To correct for library size and composition biases, normalization was performed by pooling cells and deconvoluting size factors, followed by a log2 transformation. The per-gene mean–variance relationship was modeled separately for the two tissues. Highly variable genes were selected using a 5% false discovery rate (FDR) threshold under the null hypothesis that the biological component of the gene variation is equal to zero. These genes were then used to perform a principal component (PC) analysis and only the PCs that related to the biological component of the gene variation were retained. Finally, the selected PCs were used to obtain a reduced dimensionality representation of the data by uniform manifold approximation and projection (UMAP).

A shared nearest neighbor graph-based clustering approach with 5 nearest neighbors, Jaccard index as the weighting scheme, and Louvain as the clustering method was used to identify clusters of similar cells. Cell clusters were assigned to cell types using bulk transcriptomics references of mouse cell types [39, 40]. Microglia were further annotated as homeostatic microglia (HM) and disease-associated microglia (DAM) using the marker genes described in [41].

For each cell type, pseudobulk differential expression analysis was performed, followed by gene set enrichment analysis. A neuroinflammation signature from the Human Phenotype Ontology [42] was assessed with a one-tailed Mann–Whitney *U* test.

### iMicroglia stimulation

Human iMicroglia were produced from human induced pluripotent stem cells (iPSC) with a protocol adapted from [43]. In brief, iPSC were expanded in matrigel-coated cell culture dishes and plated in ULA 96-well plates (Sigma, Switzerland) for 5 days. Embryoid bodies were then transferred to gelatin coated cell culture dishes for 20 days. CD14-positive iPSC-derived monocytes in suspension were then differentiated to iMicroglia for 20 days. For stimulation, immune complexes (ICs) were prepared fresh by combining endotoxin-free ovalbumin (Invivogen, France) and rabbit anti-ovalbumin polyclonal serum (Sigma, Switzerland) at a mass ratio of 1:6.6. The iMicroglia were plated at 25′000 cells/well in 96-well plates, incubated with serial dilutions of remibrutinib or DMSO vehicle for two hours and ICs were added. After 20 h supernatant was analyzed with anti-human TNFα HTRF (CisBio, France). Due to the variable basal response to ICs several batches of iMicroglia were compared by normalizing to the maximal TNFα secretion for each batch. Concentration responses were calculated in Prism (GraphPad, USA) using a four-parameter logistic curve model.

## Results

### Remibrutinib inhibits B cell-dependent huMOG EAE

We first investigated the efficacy of remibrutinib in the HuMOG EAE mouse model, a model in which B cells have been shown to be the dominant APC [30, 32, 44]. For this study, we selected remibrutinib doses of 3 and 30 mg/kg according to the rodent pharmacokinetic profile of remibrutinib [26]. These doses showed intermediate and near-maximal peripheral pharmacodynamic efficacy, respectively, in a preparatory BTK occupancy study at the 24 h post last dose trough timepoint (Additional file 1: Fig. S1).

**Fig. 1:**
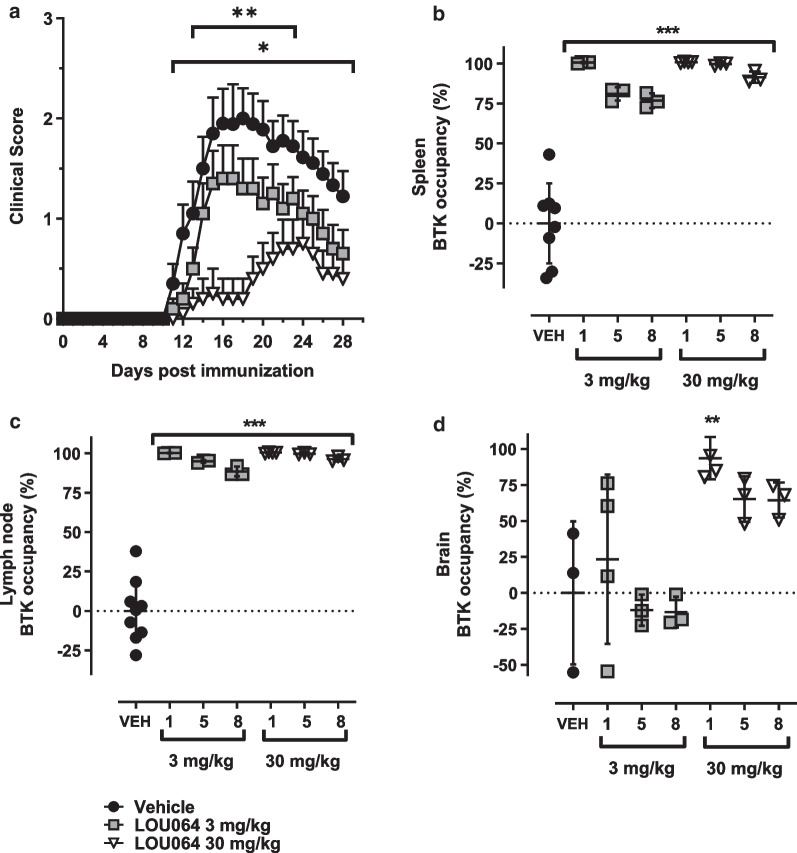
Remibrutinib inhibits HuMOG EAE. Dosing was started on day of immunization and continued to study end. **a** The 30 mg/kg dose showed statistically significant efficacy from day 11 onward (Kruskal–Wallis with Dunn’s test, *n* = 8–10 per group, means with standard errors). Peak BTK occupancy was assessed in **b** spleen, **c** lymph nodes and **d** brain homogenates 1, 5 and 8 h after the last dose. Shown are the BTK occupancy levels of individual animals and the group means with standard deviations as whiskers. Statistical significance of the 30 mg/kg dose group reached *p* < 0.05 from day 11 to study end (*) and *p* < 0.01 between days 13 and 23 (**) vs vehicle treatment (ANOVA followed by Dunnett’s test, mean ± , SEM, *n* = 5)

Remibrutinib dose-dependently reduced HuMOG EAE symptoms (Fig. 1a) and was well tolerated in all animals. The reduction of EAE scores was statistically significant for the 30 mg/kg dose group from day 11 to study end (*p* < 0.05, ANOVA followed by Dunnett’s test) and *p* < 0.01 between day 13 and day 23. At the end of the study, animals were euthanized in groups of three at one, five and eight hours post last dose to assess BTK occupancy. Peak spleen BTK occupancy was near-maximal for the 30 mg/kg group and lower for the 3 mg/kg group at these early timepoints after the last dose. A very similar result was seen in the inguinal lymph nodes (Fig. 1c). These data suggest near-maximal peripheral BTK occupancy for the 30 mg/kg dose over the twice daily dosing interval. Interestingly, brain BTK occupancy showed a stronger discrimination between the two dose levels with 3 mg/kg reaching only minimal BTK occupancy and 30 mg/kg reaching relatively high and sustained BTK occupancy after the last dose (Fig. 1d). We also determined the level of remibrutinib in blood, CSF and brain tissue for the same timepoints and found brain and CSF levels consistent with the higher brain BTK occupancy for the 30 mg/kg dose (Additional file 1: Table S1).

**Fig. 2:**
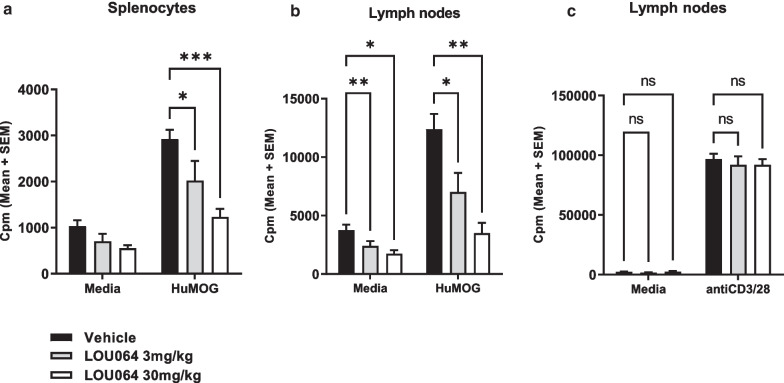
Remibrutinib inhibits huMOG specific, but not polyclonal T cell recall response. T cell recall responses were assessed 8 days after immunization with HuMOG antigen and remibrutinib dosing. Isolated splenocytes and draining lymph node cells were incubated in vitro with HuMOG for 48 h. Antigen-specific proliferation was determined by [3H]-thymidine incorporation. Remibrutinib-treated animals showed a dose-dependent significant reduction in HuMOG-induced proliferation of **a** spleen and **b** lymph node cells. **c** Polyclonal stimulation with anti-CD3/CD28 was not significantly affected by remibrutinib treatment. Statistical analysis vs vehicle treatment (ANOVA followed by Dunnett’s test (mean ± , SEM, *n* = 5, *** for *p* < 0.001, ** for *p* < 0.01 and * for *p* < 0.05)

To assess potential direct effects on immune priming and T cell activation we analyzed in a separate study splenocytes and draining lymph nodes at day 8 after immunization with HuMOG and before disease onset. Ex vivo HuMOG-specific recall responses showed a remibrutinib dose-dependent reduction of proliferation in splenic and lymph node immune cells which given the stimuli and conditions used are attributed to activated T cells (Fig. 2a and b). In contrast, plate-bound anti-CD3/CD28 polyclonal stimulation of lymph node cells was unaffected by the in vivo remibrutinib treatment (Fig. 2c). These data suggest that efficacy in the HuMOG EAE is not driven by broad antigen-independent T cell inhibition, but likely resulting from the inhibition of the initial HuMOG-specific priming of T cells that in this model has been attributed to B cells.

**Fig. 3:**
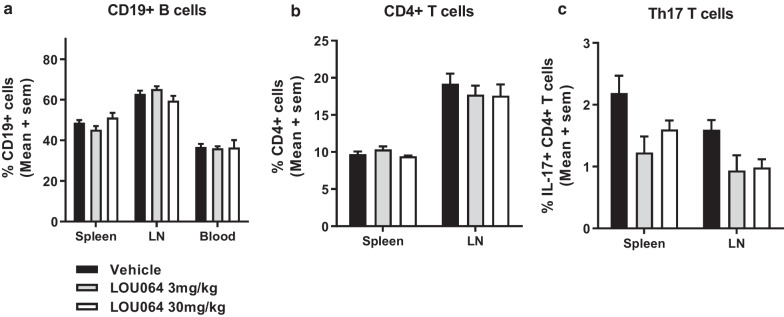
Remibrutinib does not deplete B cells in HuMOG EAE, but shows trend for reduced Th17 T cell frequencies. Mice immunized with HumanMOG showed no significant changes in the frequencies of **a** CD19 B cells or **b** CD4 T cells analyzed in the total lymphocyte gate in spleen, blood or lymphnodes (LN). In contrast, **c** PMA/ionomycin induced intracellular IL-17 staining revealed a trend for reduced Th17 CD4 T cell frequencies with a p value of 0.057 and 0.072 for 3 and 30 mg/kg dose, respectively. (Mean ± , *n* = 5, two way ANOVA followed by Dunnett’s test.)

From the same 8-day immunization study, we assessed effects of remibrutinib treatment on B cell and CD4 T cell subsets by flow cytometry. Isolated total splenocytes, lymph node cells and blood revealed no significant changes in the fraction of B cells after gating on lymphocytes (Fig. 3a), or of CD4 + T cells (Fig. 3b). Interestingly, remibrutinib treatment showed a trend for reduction in PMA/ionomycin-induced Th17 CD4 + frequencies (Fig. 3c, p = 0.057 and *p* = 0.072 for 3 and 30 mg/kg dose, respectively. *N* = 5 two way ANOVA followed by Dunnett’s test).

The total numbers of isolated cells showed no treatment effect and no changes were detected in Foxp3 + CD4 Tregs or IFNγ + CD4 Th1 cell frequencies (data not shown).

**Fig. 4:**
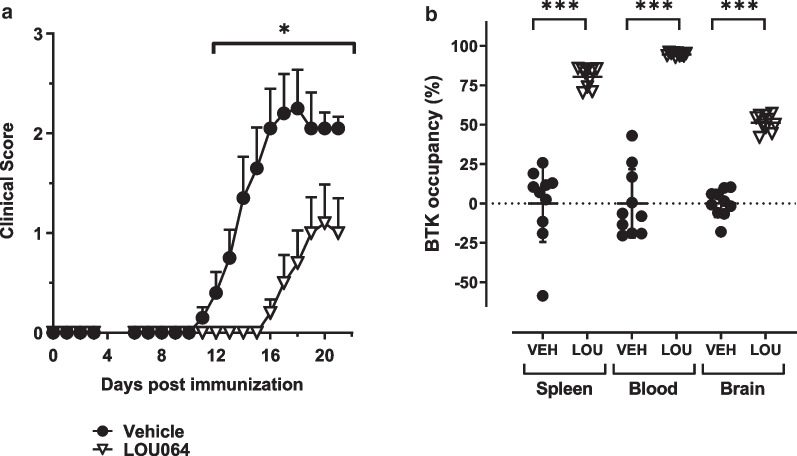
Remibrutinib inhibits RatMOG EAE. **a** Dosing of 30 mg/kg p.o. b.i.d. remibrutinib significantly reduced RatMOG EAE scores from day 12 onward (*p* < 0.05). Group sizes *n* = 10 per treatment. Statistical significance determined using Kruskal–Wallis with Dunn’s test (means with standard errors). **b** Trough BTK occupancy was assessed 16 h after the last dose in spleen, blood and brain homogenate (*p* < 0.001). Statistical significance determined by ANOVA and Sidak’s test (*LOU*  remibrutinib, *VEH* vehicle)

Having determined the efficacy of remibrutinib in the HuMOG EAE model in which B cells act as the dominant APC, we wished to assess its efficacy in the RatMOG EAE model in which B cells are not thought to be the main drivers of pathology in contrast to T cells and myeloid cells and to allow better distinction from B cell-driven effects [32]. A dose of 30 mg/kg p.o. b.i.d. significantly reduced clinical scores in the RatMOG EAE and also led to a delay in disease onset (Fig. 4a). To better describe the correlation of EAE efficacy and tissue BTK occupancy at its nadir, in this study samples were taken 16 h after the last dose. High levels of trough BTK occupancy were found in spleen, blood and brain (Fig. 4). Spleen BTK occupancy at this timepoint post last dose showed the expected reduction due to resynthesis of fresh BTK protein [26].

**Fig. 5:**
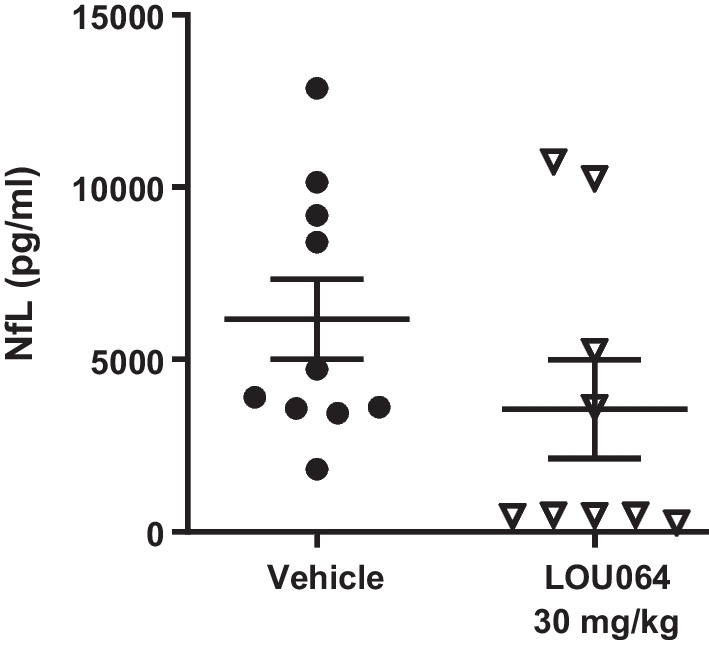
Remibrutinib reduced serum NfL levels in RatMOG EAE. Treatment with remibrutinib showed a trend for reduced serum NfL levels compared to the vehicle treatment group (means with standard errors, *p* = 0.172, *t*-test)

Of note, treatment with remibrutinib not only inhibited the EAE scores, but also showed a trend for reduction of serum NfL, a biomarker for neuroinflammation (Fig. 5). No significant inhibition of RatMOG IgG or IgM antibody response was detected (Additional file 1: Fig. S2) which is likely due to the adjuvants triggering a BTK-independent immune response.

**Fig. 6:**
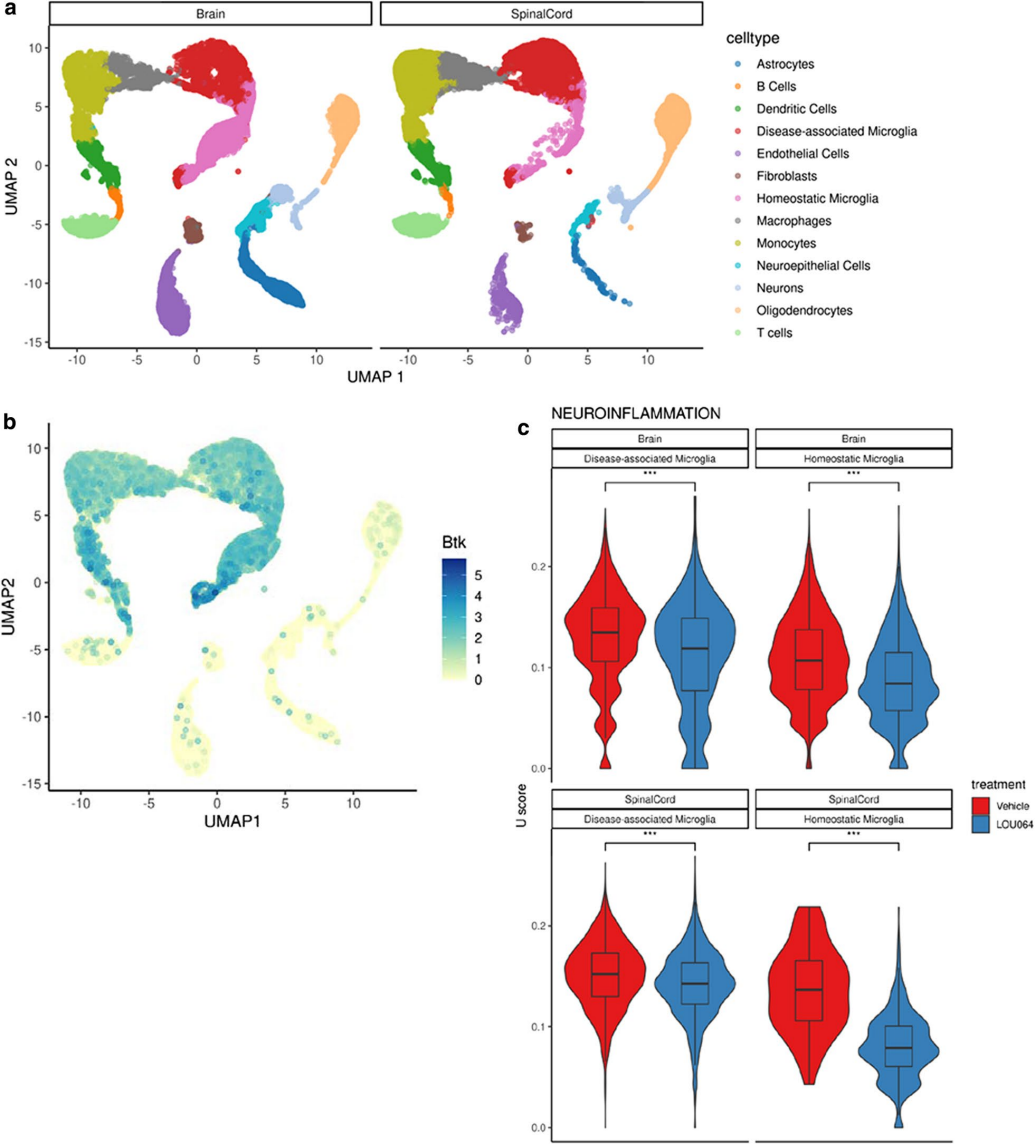
RatMOG EAE brain and spinal cord single cell gene expression and effects of remibrutinib on microglia. **a** Cell populations detected by scRNA-seq. The scRNA-seq profiling of brains and spinal cords of RatMOG EAE mice showed 13 different cell types, including stromal cells (fibroblasts, endothelial cells), all major immune cell types recruited in the CNS (B cells, T cells, DCs, monocytes and macrophages), and resident cells of the CNS: neurons, neuroepithelial cells, astrocytes, oligodendrocytes and microglia, which we further classified in HM and DAM. **b** The UMAP representation of the identified cell types in brains and spinal cords of EAE mice showed that BTK was mostly expressed in microglia, myeloid cells and B cells. **c** The analysis of the neuroinflammatory gene signature in homeostatic and disease-associated microglia showed significant effects of remibrutinib in brain and spinal cord at both timepoints (*p* < 0.001, one-tailed Mann–Whitney *U* test)

As remibrutinib did not directly inhibit T cell activation in the HuMOG model (Fig. 2c), we sought to explore the mode of action of remibrutinib in the RatMOG EAE model in more detail. In a separate RatMOG EAE study, brain and spinal cord tissue were sampled at d19 and d29 for scRNA-seq profiling. We identified 13 different cell types, including stromal cells (fibroblasts, endothelial cells), all major immune cell types recruited in the CNS (B cells, T cells, DCs, monocytes and macrophages), and resident cells of the CNS: neurons, neuroepithelial cells, astrocytes, oligodendrocytes and microglia, which we further classified in homoeostatic microglia (HM) and disease-associated microglia (DAM) (Fig. 6a). *Btk* mRNA was found to be most expressed in microglia, myeloid cells and B cells (Fig. 6b, Additional file 1: Fig. S4). Following the identification of genes differentially expressed between remibrutinib-treated animals and controls across the 13 cell types, we investigated whether signaling pathways related to immunity and inflammation were affected by remibrutinib treatment in microglia or the immune cell compartment. We observed 14 gene sets that were significantly downregulated upon remibrutinib treatment in either HM or DAM (Additional file 1: Table S2), suggesting that remibrutinib drives an anti-inflammatory effect specifically in microglia. Intrigued by these results, we tested whether a neuroinflammation signature from the Human Phenotype Ontology [42] was also affected by remibrutinib in homeostatic and disease-associated microglia, and observed a significant downregulation across most conditions (Fig. 6c).

These results point to a key role of microglial BTK in the pathogenesis of the RatMOG EAE model and show that inhibition of BTK with remibrutinib results in a significant reduction of neuroinflammatory processes in microglia.

**Fig. 7:**
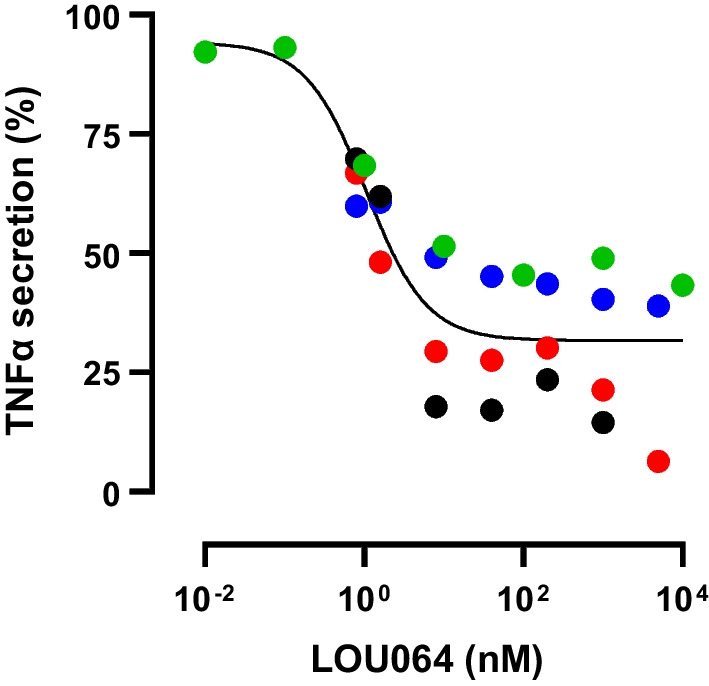
Remibrutinib inhibits IC-induced TNFα secretion from human iMicroglia in vitro*.* Four different iMicroglia batches were tested independently and showed varying levels of IC-induced TNFα (batches are color coded). Batch response was normalized to maximal TNFα secretion and concentration response curves to remibrutinib were calculated using a four-parameter logistic regression. Remibrutinib inhibited TNFα secretion with an IC_50_ of 1.1 nM

Based on this ex vivo transcriptome evidence, we assessed the direct effects of remibrutinib on in vitro cultured iMicroglia. Human iPS-derived iMicroglia were stimulated with ICs to trigger a proinflammatory reaction and secretion of TNFα (Fig. 7). While there were batch-to-batch differences in the response to ICs, as well as to the extent of remibrutinib inhibition of FcGR-induced TNFα, there was a potent concentration-dependent effect of remibrutinib with an IC_50_ of 1.1 nM (95% confidence interval 0.1091–2.945). This potency is in line with other cellular and BTK-dependent effects of remibrutinib on BCR and FcGR pathways in vitro [26].

## Discussion

The therapy of MS with disease-modifying drugs has greatly advanced with the development of injectables that deplete B cells [22]. While these drugs unveiled a central role for B cell function in the pathogenesis of MS, self-administered oral drugs that do not lead to prolonged depletion of B cells could offer an attractive and complementary treatment option to MS patients.

We first assessed the efficacy of remibrutinib in an EAE model in which the B cells are the dominant APC [30–32]. In the HuMOG EAE model, remibrutinib showed dose-dependent efficacy and while peripheral BTK occupancy was high and similar for both dose levels, central BTK occupancy was clearly higher for the 30 mg/kg dose that showed better efficacy on the EAE clinical scores. This suggests that peripheral BTK occupancy may be a less sensitive readout for EAE efficacy and higher doses leading to central BTK occupancy are required for full efficacy. The blood–brain barrier in the EAE is locally compromised due to the ongoing inflammatory process [45] and may lead to better penetration of drug into lesional areas. How these preclinical findings relate to different stages of human MS will have to be examined clinically.

The efficacy in the B cell-dependent HuMOG EAE confirms that one component of the remibrutinib mode of action in MS is the inhibition of pathogenic B cells. In the same model polyclonal T cell recall response was not inhibited suggesting that remibrutinib, owing to its excellent selectivity for BTK [26], does not directly suppress polyclonal T cell immunity even though it showed an inhibition of HuMOG-specific T cell recall proliferation, suggesting an inhibition of MOG-specific T cell priming by B cells. In addition, a trend for reduced Th17 response was found which could contribute to efficacy in an indirect manner.

To characterize B cell-independent remibrutinib pharmacology in MS, we utilized the RatMOG EAE in which remibrutinib treatment led to a reduction in disease severity and a delay in disease onset. Given the fact that remibrutinib did not directly inhibit T cells in the HuMOG immunization study, we suspected that efficacy might be driven by myeloid cells. The role of myeloid cells in the EAE models and in MS is less well established, but recently received increased attention [23, 24]. The essential role of BTK in peripheral monocytes and macrophages mediating inflammatory reactions to ICs derived from autoreactive B cells is well established [46] and it is likely contributing to efficacy in the RatMOG EAE when peripheral macrophages are recruited to the lesions.

The role of BTK in the CNS and specifically in microglia is less explored and genetic BTK deficiency in XLA patients or BTK KO mice has not been reported to lead to CNS phenotypes [13, 16]. Public scRNA-seq datasets for brain cells show a low expression of BTK mRNA in human and rodent microglia [47], and absence in other brain cells. Similarly, the present study clearly shows *Btk* mRNA present in different types of microglia in the RatMOG EAE, even if at lower levels than infiltrating B cells. At the protein level using validated specific antibodies, we and others [48] found it difficult to clearly detect BTK expression in human or rodent microglia. As this may relate to a lower abundancy of BTK protein in microglia and limited sensitivity of antibodies, we assessed functional effects of remibrutinib in the microglia EAE transcriptome. The scRNA-seq data from RatEAE brains and spinal cords clearly showed anti-inflammatory effects of remibrutinib across several pathways and cell types, including inhibition of a neuroinflammatory gene signature in microglia. This was then confirmed by a direct inhibition of FcGR-mediated TNFα secretion by remibrutinib in human iMicroglia in vitro with an IC_50_ of 1.1 nM which is in line with cellular BTK inhibition [26]. Together these data add to the evidence suggesting an important role of BTK in FcGR-mediated brain inflammation [48] and support a mechanistic facet of potential remibrutinib efficacy in MS in addition to peripheral B cell and macrophage inhibition.

Of interest, the effects of remibrutinib occurred in absence of overt B cell depletion or reduction of total Ig. While these effects may be difficult to assess in these short preclinical studies, emerging clinical data suggest that treatment of patients with BTKi like remibrutinib [28], fenebrutinib [49–51] or evobrutinib [52] may leave humoral immunity relatively untouched. This may relate to the fact that BTK is not required for survival of mature B cells [15]. Therefore, it is possible that treatment with a BTKi, after humoral immunity has developed, would not have a strong impact on B cell numbers or on total Ig levels which are largely derived from plasma cells that do not express BTK.

In conclusion, remibrutinib showed efficacy in preclinical models of MS and its mode of action extends beyond B cell inhibition to include direct anti-inflammatory effects on peripheral and central myeloid cells like microglia.

### Supplementary Information

**Additional file 1: Figure S1.** Preparatory mouse pharmacodynamic study. Based on the rodent pharmacokinetic and pharmacodynamic profile of remibrutinib [26], female C57Bl/6 mice were gavaged once daily for three days with the given doses of remibrutinib. Mice were euthanized 24 h after the last dose and trough spleen BTK occupancy was determined as described in Methods. The apparent plateau of spleen BTK occupancy is determined by the rate of fresh BTK protein synthesis once the compound has disappeared from systemic circulation until the sampling 24 h post last dose [26]. **Figure S2.** RatMOG-specific antibody response. Oral LOU064 b.i.d. treatment for 8 days did not affect MOG-specific IgM and IgG responses in serum compared to vehicle. Group sizes *n* = 4–5 per treatment, statistical significance analyzed with ANOVA (followed by Dunnett’s test). **Figure S3.** Gating strategy for intracellular cytokine analysis. The gating strategy for the flow cytometry analysis of intracellular cytokine secretion is shown based on representative FACS plots of a PMA/ionomycin-activated spleen sample. The data shown in Fig. 3c represent the difference of activated minus background of the IL17-positive population in quadrant Q1 for each mouse. **Figure S4.** Expression levels of BTK mRNA in scRNA-seq cell populations. The scRNA-seq data from RatMOG EAE brain and spinal cords revealed local BTK mRNA expression. BTK was found to be most expressed in microglia, myeloid cells and B cells. **Table S1.** Remibrutinib concentrations in HuMOG EAE. The levels of remibrutinib were determined by LC/MS [26]. The exposure in blood shows the expected levels at the 1 h timepoint with a fast decrease over the 5 and 8 h timepoints, as well as a dose-proportional increase from 3 to 30 mg/kg b.i.d. dosing. The compound levels in total brain homogenate are very low and mainly detectable at the early timepoint. Similar, but lower levels were detected in cerebrospinal fluid (CSF) and brain. Shown are averages ± SD from 4 animals for the 1 h timepoints and from 3 animals for the 5 and 8 h timepoints. **Table S2.** Pathway analysis of scRNA-seq data. Pathways and biological processes showing significant downregulation upon treatment with LOU064 in microglial cells. padj: adjusted p-value; NES: normalized enrichment score

## Data Availability

Not applicable.
