# Supplementary material for: Remibrutinib (LOU064) inhibits neuroinflammation driven by B cells and myeloid cells in preclinical models of multiple sclerosis
Source: J Neuroinflammation. 2023 Aug 26;20:194. doi: 10.1186/s12974-023-02877-9 (PMC10463946; doi:10.1186/s12974-023-02877-9)
Supplement: Supplementary file 1 — Additional file 1: Figure S1. Preparatory mouse pharmacodynamic study. Based on the rodent pharmacokinetic and pharmacodynamic profile of remibrutinib [26], female C57Bl/6 mice were gavaged once daily for three days with the given doses of remibrutinib. Mice were euthanized 24 h after the last dose and trough spleen BTK occupancy was determined as described in Methods. The apparent plateau of spleen BTK occupancy is determined by the rate of fresh BTK protein synthesis once the compound has disappeared from systemic circulation until the sampling 24 h post last dose [26]. Figure S2. RatMOG-specific antibody response. Oral LOU064 b.i.d. treatment for 8 days did not affect MOG-specific IgM and IgG responses in serum compared to vehicle. Group sizes n = 4–5 per treatment, statistical significance analyzed with ANOVA (followed by Dunnett’s test). Figure S3. Gating strategy for intracellular cytokine analysis. The gating strategy for the flow cytometry analysis of intracellular cytokine secretion is shown based on representative FACS plots of a PMA/ionomycin-activated spleen sample. The data shown in Fig. 3c represent the difference of activated minus background of the IL17-positive population in quadrant Q1 for each mouse. Figure S4. Expression levels of BTK mRNA in scRNA-seq cell populations. The scRNA-seq data from RatMOG EAE brain and spinal cords revealed local BTK mRNA expression. BTK was found to be most expressed in microglia, myeloid cells and B cells. Table S1. Remibrutinib concentrations in HuMOG EAE. The levels of remibrutinib were determined by LC/MS [26]. The exposure in blood shows the expected levels at the 1 h timepoint with a fast decrease over the 5 and 8 h timepoints, as well as a dose-proportional increase from 3 to 30 mg/kg b.i.d. dosing. The compound levels in total brain homogenate are very low and mainly detectable at the early timepoint. Similar, but lower levels were detected in cerebrospinal fluid (CSF) and brain. Shown are averages ± SD from [file 12974_2023_2877_MOESM1_ESM.docx]

**Remibrutinib (LOU064) inhibits neuroinflammation driven by B cells and myeloid cells in preclinical models of Multiple Sclerosis**

Authors

Barbara Nuesslein-Hildesheim^1^, Enrico Ferrero^1^, Cindy Schmid^1^, Catherine Huck^1^, Paul Smith^2^, Sarah Tisserand^1^, Joelle Rubert^1^, Frederic Bornancin^1^, Denis Eichlisberger^1^, Bruno Cenni^1^

Affiliations

^1^Novartis Institutes for Biomedical Research, Basel, Switzerland; ^2^Recludix Pharma, San Diego, CA

**Figure S1. Preparatory mouse pharmacodynamic study.** Based on the rodent pharmacokinetic and pharmacodynamic profile of remibrutinib (Angst *et al.*, 2020) female C57Bl/6 mice were gavaged once daily for three days with the given doses of remibrutinib. Mice were euthanized 24 hours after the last dose and trough spleen BTK occupancy was determined as described in Methods. The apparent plateau of spleen BTK occupancy is determined by the rate of fresh BTK protein synthesis once the compound has disappeared from systemic circulation until the sampling 24 hours post last dose (Angst *et al.*, 2020).


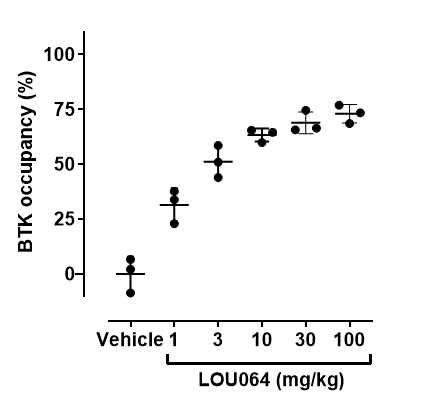


**Table S1. Remibrutinib concentrations in HuMOG EAE.** The levels of remibrutinib were determined by LC/MS (Angst *et al.*, 2020). The exposure in blood shows the expected levels at the 1 hour timepoint with a fast decrease over the 5 and 8 hour timepoints, as well as a dose-proportional increase from 3 to 30 mg/kg b.i.d. dosing. The compound levels in total brain homogenate are very low and mainly detectable at the early timepoint. Similar, but lower levels were detected in cerebrospinal fluid (CSF) and brain. Shown are averages ± SD from 4 animals for the 1 h timepoints and from 3 animals for the 5 and 8 h timepoints.

|  | 3 mg/kg | | | 30 mg/kg | | |
| --- | --- | --- | --- | --- | --- | --- |
|  | 1 h | 5 h | 8 h | 1 h | 5 h | 8 h |
| Blood (nM) | 28.2 ± 11.7 | 0.4 ± 0.04 | 0.2 ± 0.07 | 371.2 ± 149 | 19.4 ± 9.9 | 3.4 ± 2.3 |
| Brain (pmol/g) | 2.3 ± 2.2 | < 0.5 | < 0.5 | 13.5 ± 6.0 | 0.7 ± 0.02 | < 0.5 |
| CSF (nM) | 3.0 ± 3.3 | 0.5 | < 0.5 | 8.4 ± 3.1 | 0.5 | < 0.5 |

**Antibody assays**

Serum MOG-specific antibody titers were determined with sandwich immunoassays using the same recombinant MOG peptides as in the EAE immunization. Diluted serum samples were incubated for 2 hours at room temperature on MOG-coated plates (Greiner, Switzerland), plate-bound Ig were detected with subtype-specific primary detection antibodies (AbD Serotec, Switzerland) and revealed with a rabbit HRP-coupled detection antibody (AbD Serotec, Switzerland).

Total serum Ig levels were measured in the same type of assay by directly binding total serum proteins to the plates.

**Figure S2. RatMOG-specific antibody response.** Oral LOU064 b.i.d. treatment for 8 days did not affect MOG-specific IgM and IgG responses in serum compared to vehicle. Group sizes n=4-5 per treatment, statistical significance analyzed with ANOVA (followed by Dunnett’s test).


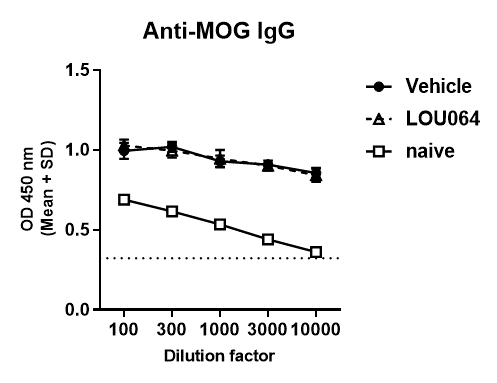

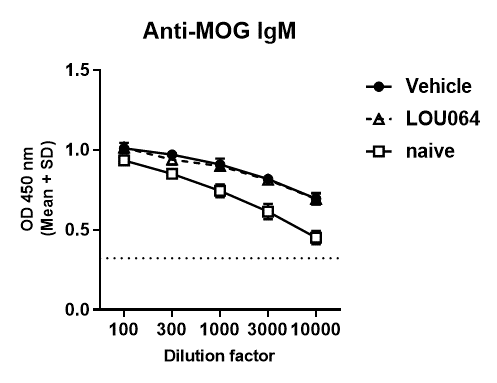


**Figure S3. Gating strategy for intracellular cytokine analysis.** The gating strategy for the flow cytometry analysis of intracellular cytokine secretion is shown based on representative FACS plots of a PMA/ionomycin-activated spleen sample. The data shown in Figure 3c represents the difference of actived minus background of the IL17 positive population in quadrant Q1 for each mouse.


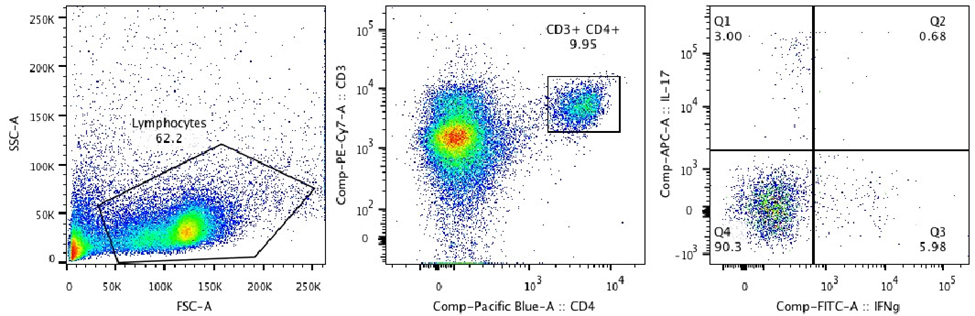


**Figure S4.** **Expression levels of BTK mRNA in scRNA-seq cell populations.** The scRNA-seq data from RatMOG EAE brain and spinal cords revealed local BTK mRNA expression. BTK was found to be most expressed in microglia, myeloid cells and B cells.


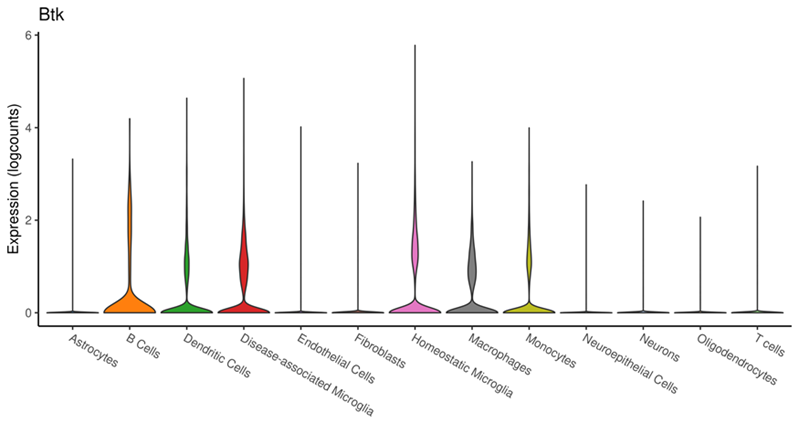


**Table S2. Pathway analysis of scRNA-seq data.** Pathways and biological processes showing significant downregulation upon treatment with LOU064 in microglial cells. padj: adjusted p-value; NES: normalized enrichment score.

| Pathway | Tissue | Time | Cell type | padj | NES |
| --- | --- | --- | --- | --- | --- |
| GOBP_INNATE_IMMUNE_RESPONSE_IN_MUCOSA | Brain | Day19 | Disease-associated Microglia | 0.004 | -1.790 |
| GOBP_INNATE_IMMUNE_RESPONSE_IN_MUCOSA | Brain | Day29 | Homeostatic Microglia | 0.001 | -1.703 |
| GOBP_HUMORAL_IMMUNE_RESPONSE_MEDIATED_BY_CIRCULATING_IMMUNOGLOBULIN | Brain | Day29 | Homeostatic Microglia | 0.001 | -1.651 |
| GOBP_HUMORAL_IMMUNE_RESPONSE | Brain | Day29 | Homeostatic Microglia | 0.001 | -1.504 |
| GOBP_INNATE_IMMUNE_RESPONSE | Brain | Day29 | Homeostatic Microglia | 0.000 | -1.460 |
| GOBP_REGULATION_OF_INNATE_IMMUNE_RESPONSE | Brain | Day29 | Homeostatic Microglia | 0.027 | -1.428 |
| HALLMARK_INFLAMMATORY_RESPONSE | SpinalCord | Day19 | Disease-associated Microglia | 0.006 | -1.413 |
| REACTOME_IMMUNOREGULATORY_INTERACTIONS_BETWEEN_A_LYMPHOID_AND_A_NON_LYMPHOID_CELL | SpinalCord | Day19 | Disease-associated Microglia | 0.042 | -1.393 |
| REACTOME_CYTOKINE_SIGNALING_IN_IMMUNE_SYSTEM | Brain | Day29 | Homeostatic Microglia | 0.001 | -1.364 |
| GOBP_ADAPTIVE_IMMUNE_RESPONSE | Brain | Day19 | Disease-associated Microglia | 0.017 | -1.323 |
| GOBP_LEUKOCYTE_MEDIATED_IMMUNITY | Brain | Day19 | Disease-associated Microglia | 0.024 | -1.266 |
| GOBP_INFLAMMATORY_RESPONSE | SpinalCord | Day19 | Disease-associated Microglia | 0.030 | -1.264 |
| GOBP_REGULATION_OF_IMMUNE_RESPONSE | Brain | Day29 | Homeostatic Microglia | 0.042 | -1.259 |
| GOBP_LEUKOCYTE_MEDIATED_IMMUNITY | Brain | Day29 | Homeostatic Microglia | 0.042 | -1.256 |
